# Golden Chanterelle or a Gold Mine? Metabolites from Aqueous Extracts of Golden Chanterelle (*Cantharellus cibarius*) and Their Antioxidant and Cytotoxic Activities

**DOI:** 10.3390/molecules28052110

**Published:** 2023-02-23

**Authors:** Nikolina Režić Mužinić, Maja Veršić Bratinčević, Marina Grubić, Roberta Frleta Matas, Martina Čagalj, Tanja Visković, Marijana Popović

**Affiliations:** 1 Department of Medical Chemistry and Biochemistry, School of Medicine, University of Split, Šoltanska 2, 21000 Split, Croatia; 2 Department of Applied Science, Institute for Adriatic Crops and Karst Reclamation, Put Duilova 11, 21000 Split, Croatia; 3 Center of Excellence for Science and Technology-Integration of Mediterranean Region (STIM), Faculty of Science, University of Split, 21000 Split, Croatia; 4 Department of Marine Studies, University of Split, Ruđera Boškovića 37, 21000 Split, Croatia; 5 Department of Pathology and Forensic Medicine, University Hospital Centre Split, 21000 Split, Croatia

**Keywords:** *Cantharellus cibarius*, edible mushroom, metabolites, phenolics, biological activity, antioxidant activity, cytotoxic activity

## Abstract

*Cantharellus cibarius*, the golden chanterelle, is the second most-collected wild edible mushroom in Europe and very frequently harvested in Croatia. Wild mushrooms have been considered a healthy food since ancient times and are today highly valued for their beneficial nutritional as well as medicinal properties. Since golden chanterelle is added to different food products to improve their nutritive value, we studied the chemical profile of aqueous extracts of golden chanterelle (at 25 °C and 70 °C) and their antioxidant and cytotoxic activities. Malic acid, pyrogallol and oleic acid were some of the main compounds identified by GC-MS from derivatized extract. *p*-Hydroxybenzoic acid, protocatechuic acid and gallic acid were the most abundant phenolics quantitatively determined by HPLC, with somewhat higher amounts for samples extracted at 70 °C. Antioxidant activity was determined by ferric reducing antioxidant power assay and oxygen radical absorption method, and the highest results were recorded for golden chanterelle extracted at 70 °C, being 41.54 ± 1.54 and 38.72 ± 2.47 µM TE/L, respectively. Aqueous extract at 25 °C showed the better response against human breast adenocarcinoma MDA-MB-231 (IC_50_ = 375µg/mL). Our results confirm the beneficial effect of golden chanterelle even under aqueous extraction conditions and highlight its significance as a dietary supplement and in the development of new beverage products.

## 1. Introduction

Edible mushrooms are one of the oldest human foods whose global production annually increases at a growth rate of around 7%. Even since ancient times, mushrooms have been considered a healthy food, and today, wild mushrooms are becoming an important part of diets because of their recognized nutritional as well as medicinal properties; they are interesting not only as tasty food rich in essential nutrients but also for their secondary metabolites with numerous biological activities [1]. *Cantharellus cibarius* Fr. (Cantarellaceae), the golden chanterelle, is a wild edible mushroom growing in forests along the Northern Hemisphere. In Europe, it is the second most-collected wild edible mushroom and is also very frequently harvested by the Croatian population [2,3]. Characteristic composition of mushrooms may be associated with environmental and geographical factors [4,5]. In general, mushrooms represent a valuable constituent of the daily diet since they are rich in carbohydrates (polysaccharides, glucans, galactans and mannans), proteins, polyunsaturated fatty acids (PUFA), secondary metabolites (phenolics, vitamins, terpenoids and indole compounds) and microelements (zinc, copper, iron and selenium). These natural compounds could be exploited in various fields and are currently greatly explored and used for health-promoting mushroom-based products [6]

*Cantharellus cibarius* is rich in saturated (SFA), monounsaturated (MUFA) and polyunsaturated (PUFA) fatty acids. The ratio of MUFA/SFA and PUFA/SFA is 1.6 and 3, respectively, making the golden chanterelle a valuable source of favorable fatty acids. Linoleic acid is the most represented PUFA in *C*. *cibarius*, yielding up to 54% [4]. Golden chanterelle is also rich in essential amino acids, especially threonine and lysine [2], as well as in serotonin, kynurenine sulfate and 5-hydroxytryptophan [7]. It is also a good source of vitamin D and ergosterol [8]. Several authors report homogentisic acid and gallic acid as the most abundant phenolics, followed by pyrogallol, gentisic acid and protocatechuic acid [5,9].

Numerous biologically active compounds, some of which are PUFA, indolic compounds, vitamin D, phenolics and zinc, are responsible for the anti-inflammatory properties of the chanterelle [5,6]. Mushroom extracts contain many components with specific biological effects, and a majority of edible mushrooms possess antioxidant activity [10]. Because of its antioxidant potential, *C. cibarius* is being used more and more in various edible products to conserve the product and prevent microbial growth [11]. Recently, mushrooms have become an important potential natural agent for the prevention and help in treatment of numerous diseases [12]. 

Moreover, as the beneficial properties of *C. cibarius* are being recognized, this mushroom is more and more becoming a healthy addition to coffee and similar products. The direct or indirect use of mushrooms in processed food is increasing due to their health benefits or the need of an increase product quality. Thus, various types of mushrooms can be found as functional ingredients in many bakery products, primarily in bread, in meat products, as flavor enhancers in soups or seasoning or as a source of fermentation in the beer and wine production [13].

Due to the fact that *Cantharellus cibarius* is one of the most frequently collected and used wild mushrooms in Croatia and recently is more consumed as a beverage additive, the aim of the study was to determine metabolite profiles of aqueous extracts at different temperatures (25 °C and 70 °C, i.e., cold and hot beverage extraction) as well as to determine their antioxidant and cytotoxic activities. 

## 2. Results and Discussion

The chemical composition of aqueous extracts from *Cantharellus cibarius* (CC) was studied through individual phenolic compounds determination by U*V/V*IS-HPLC, as well as through metabolite profiling performed by trimethylsilyl derivatization of extract following GC-MS analysis.

### 2.1. Phenolic Analysis

The individual profile of eight phenolic compounds was obtained using high-performance liquid chromatography coupled to U*V/V*IS detector (Figure S1) for CC extracts at 25 °C and 70 °C. Phenolic compounds, five phenolic acids (gallic, protocatechuic, *p*-hydroxybenzoic, caffeic and ferulic acids) and one flavonoid (myricetin), occurring in water extract of CC were confirmed and quantified by comparison of the absorption spectrum at 220 and 320 nm with the retention time of phenolic compound standards. Two flavonoids, rutin and quercetin, were detected but not quantified.

Phenolic acids, which represent the main phenolic components in edible mushrooms, can be divided into two main groups—hydroxybenzoic and hydroxycinnamic acids—of which, among others, the most abundant are from hydroxybenzoic class, namely, *p*-hydroxybenzoic, protocatechuic and gallic acid [14]. These observations coincide with the results of our study (Table 1), where *p*-hydroxybenzoic acid was the most abundant phenolic component in both extracts (2.38 ng per 100 g DW for extracts at 70 °C and 1.98 ng per 100 g DW for extracts at 25 °C), followed by protocatechuic (1.02 ng per 100 g DW for extracts at 70 °C and 0.97 ng per 100 g DW for extracts at 25 °C) and gallic acid (0.86 ng per 100 g DW for extracts at 70 °C and 0.83 ng per 100 g DW for extracts at 25 °C). Islam et al. [15] proved the presence of gallic acid as a major phenolic compound in *C. cibarius* hydro-acetonic acid. Contrary to our results, Barros et al. [16] examined phenolic acids in 16 different Portuguese wild mushrooms, including protocatechuic and *p*-hydroxybenzoic acid, but did not prove the presence of any tested phenolic compound in *Cantharellus cibarius*. This could be due to harvesting time, extraction solvent and extraction procedure as well as the analytical method used for phenolic compound determination. The importance of the presence of phenolic acid is manifested in the protection of plant and fungal cells from UV radiation, insect and microbial damage [17,18]. 

Ferulic and caffeic acids were qualitatively and quantitatively determined in both CC extracts, which together with *p*-coumaric, *o*-coumaric and caffeoylquinic acid derivatives make up the majority of identified cinnamic acid derivatives in edible mushrooms. Among the 16 different mushroom species, Dimopoulou et al. [19] reported the highest concentration of caffeic acid in *Cantharellus cibarius* at a value of 16.34 μg per g DW. 

Rutin, quercetin and myricetin were analyzed in water extracts of CC as representatives of flavonoids, and only myricetin was quantified; while the other two were identified, we were unable to quantify them. Rutin and quercetin were not quantified, but their presence was determined, probably due to the low concentration of the compounds present in the samples (concentration below the detection limit), as well as due to the method and instrument limitations. The same concentration values were obtained for myricetin in both extracts prepared at different temperatures, 0.47 ng per 100 mg DW. While measuring the individual concentrations of phenolic acids, a higher value was obtained for each individual phenolic acid in the extracts prepared at 70 °C except for gallic acid. 

Flavonoids represent an important group of phenolic compounds and are often associated with plants. However, edible mushrooms also seem to represent their potential source, although this was debatable since it was considered that only plants have the biosynthetic ability to produce flavonoids. However, the concentration may vary depending on the type of mushroom, environmental factors and genetic predispositions [14,20,21]. A study by Kozarski et al. [21] on the methanol extract of CC showed that flavonoids, along with phenols (which were the most abundant antioxidant compounds), made up 86% of the total phenol content. Butkhup et al. [22] studied bioactivity and phenolic contents of methanol extracts of twenty-five wild edible mushrooms from Thailand, including *Cantharellus cibarius*. In contrast to our results, quercetin (0.24 g per kg DW) was found in a higher quantity than myricetin (0.03 g per kg DW). 

### 2.2. Derivatized Extract Analysis

**Figure 1:**
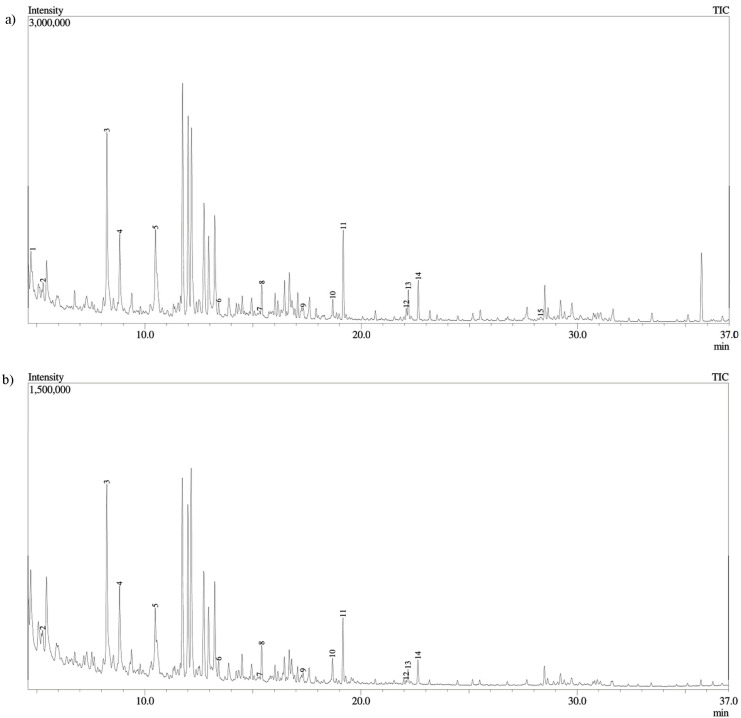
GC-MS chromatogram of *Cantharellus cibarius* (**a**) aqueous extract at 25 °C, (**b**) aqueous extract at 70 °C, after derivatization with BSTFA. Peak assignment is given in Table S1.

To further explore metabolite profile of golden chanterelle, evaporation of aqueous extract followed by derivatization with bis(trimethylsilyl)trifluoroacetamide (BSTFA) was performed. A total of fifteen compounds were identified by gas chromatography-mass spectrometry (Figure 1, Table S1), among which four belong to a chemical class of saturated fatty acids (SFA), three to a class of dicarboxylic acids, and two to a class of monounsaturated (MUFA). Among other interesting compounds, pyrogallol and linoleic acid were identified. 

Fatty acids have both a positive and a negative role in the prevention and treatment of diseases as well as in maintenance of human health. High dietary intake of SFA is associated with increase in low-density lipoproteins (LDL) cholesterol and numerous diseases, whereas replacement of SFA with unsaturated FA, PUFA in particularly, reduces cardiovascular diseases [23]. Determination of the PUFA/SFA ratio of is important in assessment of nutritional and medicinal value of food products. We have identified four SFA (myristic, pentadecanoic, palmitic and stearic acid) in *Cantharellus cibarius* aqueous extract, two MUFA (palmitelaidic acid and oleic acid) and one essential PUFA, linoleic acid. Galgowska et al. [4] determined the fatty acid profile in three mushroom species, golden chanterelle included, as well as the percentage ratio of SFA, MUFA, PUFA, UFA and lipids. The highest percentage of PUFA was found in *C. cibarius*, with a beneficial percentage ratio PUFA/SFA of 3.07, thus making a golden chanterelle an excellent nutritional product for the maintenance of cardiovascular health. Panchak et al. [24] studied hexane extract of *C. cibarius* and determined the highest level of linoleic acid (31.42%), followed by oleic (17.92%) and palmitic acid (11.59%), while stearic acid was not detected.

Organic acids are one of the factors known to contribute to organoleptic characteristics of food products. We have identified four organic acids, two of which (malic and citric acid) were previously found in *C. cibarius*. Malic acid was present in the highest amount, followed by citric acid [25,26]. Other previously reported organic acids present in smaller amount were ascorbic, acetic, fumaric, formic and shikimic acid. 

One phenolic compound, pyrogallol, was also identified in the *C. cibarius* aqueous extract. The standard of pyrogallol was not assessable to confirm this finding by HPLC, but several authors did confirm its presence in *C. cibarius* extracts [9,18].

The amount of phenolic in aqueous extract of *C. cibarius*, as well as the presence of other biologically important metabolites identified (MUFA, linoleic acid and organic acids) points to the beneficial effect of this mushroom not only as a food product but also as a beverage additive. Keeping in mind that golden chanterelle is also rich in polysaccharides [27], aqua-soluble beta glucan, in particular, additional beneficial effect of the extract is to be expected. Beta glucans have significant effects on various branches of the immune system and are able to significantly improve human health [28]. The percentage of beta glucan in analyzed *C. cibarius* was 13.11% of DW, while alpha-glucans were 0.7% of DW (results not shown).

### 2.3. Antioxidant Activity 

Two antioxidant assays, FRAP and ORAC, were performed to determine the antioxidant capacity of *Cantharellus cibarius* aqueous extracts. Usually, a few methods based on different principles and action techniques are used to assess the antioxidant capacity of extracts, given that there is no single method or official standardized method for the assessment of antioxidant capacity [29,30]. The reduction activity of extracts was assessed using the FRAP method, in which higher Fe(III) reduction is associated with a higher antioxidant capacity. The oxygen radical absorption method (ORAC) has found its place among a large number of developed, documented and reviewed methods for measuring antioxidant capacity. This method, with some adjustments, is widely used to determine the antioxidant activity of food additives and a large number of plant extracts. The results are presented in Table 2.

Both assays confirmed the antioxidant activity of *Cantharellus cibarius* extracts prepared under different temperatures, 25 and 70 °C. Almost two-fold higher antioxidant activity for both methods was measured for extracts that were prepared at the higher temperature. This could be due to the higher number of phenolic acids extracted, as shown in Table 1. Shah and Modi [31] examined the antioxidant activity of water extracts of three types of mushrooms using three different antioxidant assays, including FRAP, determined that the results obtained via FRAP assay were reproducible for all concentrations and concluded that FRAP is a suitable method for determining antioxidant activity. Witkowska et al. [32] studied sixteen edible species of wild-growing mushrooms to test their antioxidant activities, including *Cantharellus cibarius*, and reported the FRAP value of 1.47 mmol per 100 g of dry mass mushroom content. Piljac-Žegarac et al. [33] determined the antioxidant activity of three wild Croatian mushrooms and four cultivated mushroom varieties using FRAP assay in methanol and water extracts. The water extracts gave better results, from 1.87 to 47.75 mmol Fe^2+^ per kg of extract. The comparison of results among different studies is difficult as data are expressed in different units.

Comparing the obtained results with those from our study, *Cantharellus cibarius* extracts gave higher results than all three examined wild mushrooms and two cultivated ones. Contrary to our results, several authors in their studies have proven that the boiling process affects the reduction of antioxidant activity, as well as the content of polyphenols in different mushroom varieties [34,35]. Kettawan et al. [29] studied the antioxidant activity in ten raw and boiled edible mushrooms using FRAP, ORAC and DPPH assays. Both FRAP and ORAC gave better results in raw samples, suggesting that higher temperatures significantly decrease the polyphenol content and antioxidant activity in all tested samples. Additionally, by comparing the obtained results, both assays gave better results when testing the antioxidant activity at a higher temperature for extracts prepared at a temperature of 70 °C.

### 2.4. Cytotoxic Activity 

**Figure 2:**
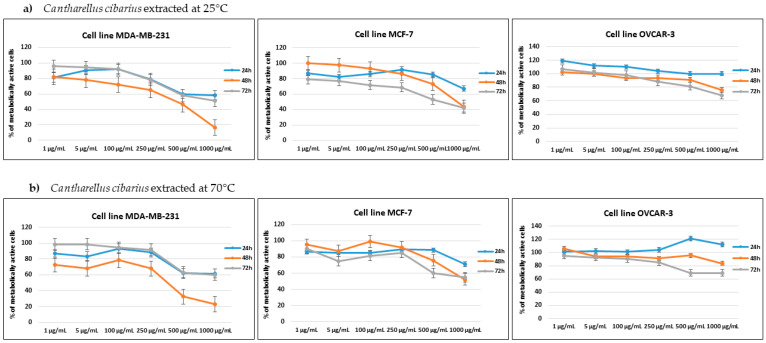
Percentage of metabolically active human breast adenocarcinoma MDA-MB-231, human breast metastatic adenocarcinoma MCF7 and human ovarian carcinoma OVCAR-3 cell lines after 24, 48 and 72 h of incubation with different concentrations of (**a**) *Cantharellus cibarius* mushroom extracts at 24 °C and (**b**) *Cantharellus cibarius* mushroom extract at 70 °C.

**Figure 3:**
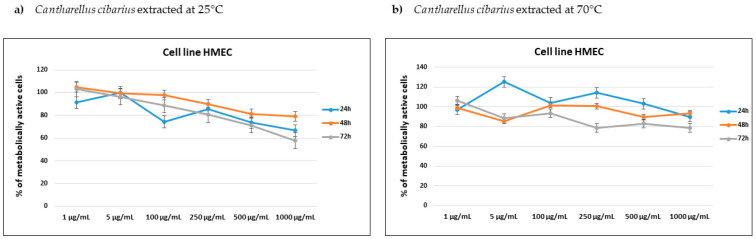
Percentage of metabolically active human mammary epithelial HMEC cell lines after 24, 48 and 72 h of incubation with different concentrations of (**a**) *Cantharellus cibarius* mushrooms extracts at 24 °C and (**b**) *Cantharellus cibarius* mushrooms extract at 70 °C.

The cytotoxic effect of *Cantharellus cibarius* mushroom extract was tested using an MTT assay against four cell lines: human breast adenocarcinoma MDA-MB-231, human breast metastatic adenocarcinoma MCF7, human ovarian carcinoma OVCAR-3 and human mammary epithelial HMEC cell lines. The percentage of metabolically active cells for tested concentrations after 24 h, 48 h and 72 h incubation are shown in Figure 2 and Figure 3. 

The aqueous mushroom extract (25 °C and 70 °C) did not reach the IC_50_ value for any tested concentration on the OVCAR-3 cell line and had similar activity for both extract temperatures. Both aqueous extracts showed the best results: 68–70 % of metabolically active cells after 72 h. Aqueous mushrooms extracts (25 °C and 70 °C) showed the best results for human breast adenocarcinoma MDA-MB-231, where IC_50_ was reached after 48 h of incubation (IC_50_ = 375 μg/mL for both measurements). Mushroom extract at 25 °C also reached IC_50_ in a concentration of 750 μg/mL after 48 h and 72 h on MCF-7, while mushroom extract at 70 °C was close to that cytotoxic activity and almost reached IC_50_ in the concentration of 1000 μg/mL after 48 and 72 h. The aqueous mushroom extract (25 °C and 70 °C) did not reach the IC_50_ value for any tested concentration on the HMEC cell line, implying that the mushroom extract is less potent in regard to normal cells, in contrast to breast adenocarcinoma cell lines. Therefore, the aqueous extracts of golden chanterelle have cytotoxic activity against MDA-MB-231 and MCF-7 cell lines.

Mushrooms have been used as food products for culinary purposes since ancient time but today are becoming more popular as a remedy for several medical conditions, including cancer. Mushroom extracts are also studied as an addition to cancer therapy, as a combination of biologically active natural compounds and chemotherapy, with a positive effect on treatment efficacy and improvement of patients comfort and safety [36]. Extracts of numerous of mushroom species have been tested for anticancer activities, resulting in reducing the viability of over 38 different cancers. From all cancer tested, breast cancer proved to be most sensitive to mushroom extract; 61 extracts studied showed strong antiproliferative potential [37]. To the authors’ best knowledge, this is the first report of *Cantharellus cibarius* aqueous extract impacting human breast adenocarcinoma and ovarian carcinoma cell lines. CC aqueous extracts on MDA-MB-231 and MCF7 cell lines showed antiproliferative potential, especially for the extraction at 25 °C. Since phenolic compounds were extracted slightly in higher amount at 70 °C and the profile of derivatized metabolite from the extract was similar for extraction at 25 °C and 70 °C, authors can only hypothesize that higher temperature has an effect on the difference in composition of some other compound from the extract or that the synergistic effect of compounds from the extract at 25 °C is higher than that of the compounds extracted at 70 °C. Mushroom extracts are also rich in polysaccharides that have antitumor and immunomodulatory affect; however, they do not attack cancer cells directly, needing instead of activate different immune responses in the host through T-cells [38]. Kozarski et al. [21] studied methanol extract of CC against MDA-MB-231 and obtained similar IC_50_ = 0.307 mg/mL as we did from aqueous extracts (IC_50_ = 375 μg/mL). They also studied cytotoxicity against several other cell lines and found that CC methanol extract had highest cytotoxicity against human myelogenous leukemia K562 cells but was much less active against nontumorous cell lines (human fetal lung fibroblasts MRC-5, IC_50_ = 0.661 mg/mL and human lung bronchial epithelial cells BEAS-2B, IC_50_ = 0.539 mg/mL), indicating anticancer activity of the extract. Lemieszek et al. [39] studied the effect of ethanol insoluble and water-soluble small RNA fraction, purified from coextracted polysaccharides, and revealed that small RNA from *C. cibarius* has strong antiproliferative activity against human colon adenocarcinoma HT-29 (IC_50_ of 1.9 μg/mL) and LS180 (IC_50_ of 5.6 μg/mL) cell lines. Overall, *Cantharellus cibarius* extracts are potentially a great source of biologically relevant compounds against various cancer types.

## 3. Materials and Methods

### 3.1. Chemicals

Standards were purchased as follows: caffeic acid, quercetin, protocatechuic acid, *p*-hydroxybenzoic acid, rutin, myricetin and alkane series were purchased from Sigma—Aldrich (St. Louis, MO, USA); gallic acid from Acros Organics (Geel, Belgium); and ferulic acid from Fluka Chemie (Buchs, Switzerland). The derivatization reagent N,O-Bis(trimethylsilyl)trifluoroacetamide (BSTFA) was purchased from Sigma–Aldrich (St. Louis, MO, USA), phosphoric acid was purchased from Honeywell Fluka (Charlotte, NC, USA) and acetonitrile and methanol were purchased from VWR (Radnor, PA, USA).

### 3.2. Cantharellus Cibarius Aqueous Extract

The powder extract of *Cantharellus cibarius* was obtained by a local provider (Mushroomcups, Solin, Croatia). The fine mushroom powder was dissolved in deionized water (1 mg/mL) at temperatures of 25 and 70 °C to simulate average hot and cold beverage extraction (if mushroom powder was added to, i.e., juice or coffee). After 1 min of stirring, the aqueous extraction was tempered at room temperature. Afterward, an aliquot of the extracts was centrifuged for 2 min at 1000 rpm (Eppendorf centrifuge 5702, Hamburg, Germany). Aqueous extractions were stored at 4 °C until analysis.

### 3.3. HPLC Conditions for Phenols Determination

The HPLC used for phenolic compounds in *Cantharellus cibarius* extracts was a Shimadzu LC-20 series with U*V/V*IS detector (Shimadzu, Japan). Phenolic compounds were separated using Shim-pack GIST C18 column (250 × 4.6 mm, 5 µm; Shimadzu, Japan), and temperature was maintained at 35 °C. The mobile phase flow rate was 1 mL/min, consisting of mobile phase A (ultrapure grade water/o-phosphoric acid, 99.8:0.2, *v/v*) and mobile phase B (methanol/acetonitrile, 1:1, *v/v*). Run time was 65 min, and gradient was applied as follows: initial 4% B, 15% B up to 16 min and maintained until 37.5 min, 35% B to 50 min and maintained until 60 min, and 4% B until 62 min to the end of analysis at 65 min. In order to validate the method, calibration curves using 6 calibration levels ranging from 0.1 to 5 mg/L were used for each tested phenolic compound. In addition, the coefficient of determination R^2^, slope and intercept, as well as detection and quantification limits, were determined (Table S2). Samples were filtered through 0.45 um PVDF filters before injection into HPLC. Samples were injected in quadruplicate in volume of 20 µL.

### 3.4. Extract Derivatization 

*Cantharellus cibarius* aqueous extract was evaporated and derivatized according to Frleta et al. [40] with some small adjustments. Aqueous extracts (1 mL) were evaporated at the room temperature in centrifugal evaporator (RC10-22, Jouan, Herblain, France) until the samples were dried. Bis(trimethylsilyl)trifluoroacetamide (BSTFA) was used as derivatizing agent; 30 μL was added at 20 °C to the dried extracts for 20 min prior the analysis.

### 3.5. GC-MS Conditions for Derivatized Extracts 

BSTFA derivatized extracts were analyzed by gas chromatography-mass spectrometry (GC-MS) with Shimadzu (Kyoto, Japan) Nexis GC-2030 gas chromatograph equipped with an automatic liquid injector model AOC-20i coupled with a Shimadzu QP2020 NX mass detector. The samples were analyzed on the SH-5MS column (30 m length; inner diameter, 0.25 mm; and stationary phase layer thickness, 0.25 µm; Shimadzu, Kyoto, Japan) using ultrapure helium as a carrier gas, with flow rate at 2.46 mL min^−1^. The volume of the injected sample was 1 μL, split ratio 1:10, and the inlet temperature was set at 280 °C. The initial column temperature was set at 120 °C for the first 3 min, increased to 292 °C at a rate of 5 °C min^−1^, then increased to 320 °C at a rate of 30 °C min^−1^ and maintained as isothermal for 17 min. The analysis was performed with MS full scan 35–750 *m*/*z* [41]. Identification of derivatized compounds was performed by comparing their derivative mass spectra (TMS) and GC retention indices with series of n-hydrocarbons, as well as by the computer matching with commercial libraries (Wiley 12 and NIST 2020). Derivatized extracts were analyzed in duplicate. 

### 3.6. Antioxidant Activity 

#### 3.6.1. Ferric Reducing Antioxidant Power (FRAP) Assay 

The ferric reducing/antioxidant power (FRAP) of *Cantharellus cibarius* extract was determined according to Benzie and Stain [42] with slight modifications. FRAP was used to measure the reducing activity of extracts. Briefly, 10 μL of samples or the Trolox (6-hydroxy-2,5,7,8-tetramethylchroman-2-carboxylic acid) standard solution was added to 300 μL of freshly prepared and mixed completely. The FRAP reagent solution was added to the microplate wells, and the absorbance was measured at 592 nm; then 10 μL of the sample was added to the FRAP reagent, and after 4 min, the change in absorbance was measured. The change in absorbance was compared with the values measured for standard Trolox solutions and was calculated as the difference between the final absorbance value of the reaction mixture 4 min from the beginning of the reaction and the absorbance of the FRAP reagent before sample addition. Samples were diluted, and the results were expressed as μM of Trolox Equivalents per L of extract (μM TE/L).

#### 3.6.2. Oxygen Radical Absorbance Capacity (ORAC) Assay 

Extracts’ ability to scavenge peroxyl radicals was measured using ORAC assay [43]. Briefly, 25 μL of the mushroom extract was incubated with 150 μL of fluorescein (in 1:10 dilution) at 37 °C for 30 min. Afterward, 25 µL of freshly prepared 2,2′-Azobis 2-amidinopropane dihydrochloride (AAPH) was instantly added, and measurements were performed at 485 and 520 nm for 80 min (every 60 s). Trolox was used as a positive control. Samples were analyzed in triplicate, and the results were expressed as μM TE/L of extract. 

### 3.7. Cytotoxic Activity 

In order to determine cytotoxic activity of *Cantharellus cibarius* mushroom extract, cell viability assay (3-(4,5-dimethylthiazol-2-yl)-2,5-diphenyltetrazolium bromide, MTT) was performed on four cell lines: human breast adenocarcinoma MDA-MB-231, human breast metastatic adenocarcinoma MCF7, human ovarian carcinoma OVCAR-3 cell line (LGC Standards) [44] and human mammary epithelial HMEC cell line (ATCC). 

MDA-MB-231, MCF7, OVCAR-3 and HMEC cell lines were seeded and incubated overnight adherence in 96-well plates at a density for MDA-MB-231 of 10,000 cells/well, MCF7 of 8000 OVCAR-3 of 6500 and HMEC of 4000, followed by incubation with medium alone or with serial dilutions extracts at concentrations in a range 1–1000 µg/mL for 24, 48 and 72 h (in triplicate). Afterward, 100 μL of 0.5 g MTT/L was added to each well, mixed and incubated at 37 °C for 2 h; the medium was removed, and 10% dimethylsulfoxide (DMSO) was added for another 10 min at 37 °C. The indicator of metabolically active cells formazan was formed and measured at 570 nm on the microplate reader (BioSan, Riga, Latvia). The calculation of IC50 values was performed with Microsoft Excel 2016. To determine the differences between tested concentration, analysis of variances one-way ANOVA was performed using Past 3.X software (version 3.14, University of Oslo, Norway), with the significance level at *p* < 0.05. 

## 4. Conclusions

This study aimed to chemically characterize aqueous extracts of *Cantharellus cibarius* at different temperatures that simulated average hot and cold beverage extraction, as well as to determine their antioxidant and cytotoxic properties. Our results acknowledged golden chanterelle extract as a good source of phenolic compounds, as well as organic acids, MUFA and linoleic acid. Antioxidant activity of both extracts was confirmed by two assays (FRAP and ORAC), as well as cytotoxicity against human breast adenocarcinoma MDA-MB-231 cell line, while the extract at 25 °C showed better response against human breast metastatic adenocarcinoma MCF-7 cell line.

Since *Cantharellus cibarius* has a beneficial effect even under aqueous extraction conditions, our findings confirm its significance as a dietary supplement and nutraceutical extract but also directly in the development of new beverage products that could be organoleptic and nutritionally appealing.

## Figures and Tables

**Table 1:** Phenolic compounds from *Cantharellus cibarius* aqueous extract at 25°C and 70 °C.

Phenolic Compound	25 °C		70 °C		Concentration Ratio (25 °C/70 °C)
	Concentration (mg/L)	Percentage (%)	Concentration (mg/L)	Percentage (%)	
Gallic acid	0.83 ± 0.00	1.01 ±0.02	0.86 ± 0.00	1.08 ± 0.02	0.97
Protocatechuic acid	0.97 ± 0.00	1.05 ± 0.04	1.02 ± 0.04	1.25 ± 0.02	0.95
*p*-Hydroxybenzoic acid	1.98 ± 0.02	0.85 ± 0.08	2.38 ± 0.08	1.02 ± 0.02	0,83
Caffeic acid	0.21 ± 0.00	0.9 ± 0.03	0.23 ±0.01	0.89 ± 0.01	0.91
Ferulic acid	0.28 ± 0.00	2.27 ± 0.15	0.39 ± 0.01	2.22 ± 0.1	0.72
Quercetin	tr.	-	tr.	-	-
Rutin	tr.	-	tr.	-	-
Myricetin	0.47 ± 0.00	0.28 ±0.01	0.47 ± 0.02	0.31 ± 0.00	1

Concentration of phenolic is expressed as mean ± SD. The values are represented as ng/100 mg of *Cantharellus cibarius* fine powder. Component difference percentage is expressed as %. tr.—trace amounts.

**Table 2:** Antioxidant activity of *Cantharellus cibarius* aqueous extract at 25 °C and 70 °C.

Antioxidant Assay	*Cantharellus cibarius* Aqueous Extract	
	25 °C	70 °C
ORAC (μM TE/L)	19.71 ± 0.64	38.72 ± 2.47
FRAP (μM TE/L)	22.82 ± 0.94	41.54 ± 1.54

Results are expressed as mean ± SD. In ORAC assay, 1:10 dilution of aqueous extract was used.

## Data Availability

All data are included within the article and in Supplementary Materials.

## Supplementary Materials

The following supporting information can be downloaded at: https://www.mdpi.com/article/10.3390/molecules28052110/s1, Table S1: TIC normalized peak area % of identified compounds (at 25 °C and 70 °C) and MS spectra of corresponding trimethylsilyl (TMS) metabolites from Cantharellus cibarius aqueous extracts; Table S2: Validation parameters for phenolic compounds; Figure S1: HPLC chromatograms of golden chanterelle aqueous extracts for extraction at 70 °C, at 220 (black line) and 320 nm (pink line). Identified and quantified phenolic compounds in order of appearance with specified retention times: gallic acid (8.82 min), protocatechuic acid (14.27 min), p-hydroxy benzoic acid (20.58 min), caffeic acid (26.26 min), ferulic acid (47.32 min), quercetin (50.58 min), rutin (51.57 min) and myricetin (55.84 min).

## Sample Availability

Samples of the *Cantharellus cibarius* mushroom extract are available from the authors.
