# Supplementary material for: Golden Chanterelle or a Gold Mine? Metabolites from Aqueous Extracts of Golden Chanterelle (Cantharellus cibarius) and Their Antioxidant and Cytotoxic Activities
Source: Molecules. 2023 Feb 23;28(5):2110. doi: 10.3390/molecules28052110 (PMC10004332; doi:10.3390/molecules28052110)
Supplement: Supplementary file 1 [file molecules-28-02110-s001.zip › molecules-2132298-supplementary.pdf]

Table S1. TIC normalized peak area % of identified compounds (at 25 °C and 70°C) and MS spectra of corresponding trimethylsilyl (TMS) metabolites from *Cantharellus cibarius* aqueous extracts.

	RI	Compound	25°C	70°C	m/z
1	1319	Butanedioic acid, 2TMS derivative	1.39	4.13	147(100), 73(30), 148(15), 75(15), 247(14), 341(9), 149(9), 55(8), 45(6), 56(6)
2	1351	(*E*)-2-Butenedioic acid, 2TMS derivative	0.47	0.57	245(100), 147(35), 73(24), 246(21), 75(18), 143(16), 247(9), 45(8), 133(7), 115(6)
3	1506	Malic acid, 3TMS derivative	7.70	8.41	73(100), 147(52), 233(24), 245(11), 133(9), 75(9), 74(9), 189(8), 148(8), 55(8)
4	1535	Pyroglutamic acid, 2TMS derivative	3.14	3.65	156(100), 73(51), 147(13), 157(13), 258(9), 230(8), 45(7), 75(5), 74(5), 158(4)
5	1617	Pyrogallol, 3TMS derivative	1.71	1.52	342 (100), 73 (93), 239(75), 343(32), 45(18), 240(17), 344(16), 241(9), 327(8), 254(7)
6	1754	Xylitol, 5TMS derivative	0.39	0.52	73(100), 217(84), 103(51), 147(43), 205(30), 307(26), 129(24), 319(22), 218(20), 117(14)
7	1849	Citric acid, 4TMS derivative	0.08	0.03	73(100), 273(96), 147(61), 274(22), 363(21), 347(21), 375(21), 211(13), 75(11), 148(11)
8	1853	Myristic acid, TMS derivative	0.86	0.88	117(100), 285(75), 73(72), 75(58), 132(45), 129(34), 145(21), 55(18), 286(16), 43(16)
9	1952	Pentadecanoic acid, TMS derivative	0.29	0.30	117(100), 299(69), 75(57), 132(53), 129(33), 145(25), 43(22), 55(20), 73(18), 131(15)
10	2029	Palmitelaidic acid, TMS derivative	0.47	0.57	73(100), 117(91), 75(84), 129(62), 311(60), 55(45), 43(28), 145(28), 96(27), 41(26)
11	2050	Palmitic Acid, TMS derivative	2.54	1.79	117(100), 73(81), 313(64), 75(56), 132(51), 129(36), 145(26), 43(21), 55(19), 314(15)
12	2215	Linoleic acid, TMS derivative	0.31	0.09	75(100), 73(87), 67(65), 81(54), 337(54), 95(51), 55(46), 117(33), 96(33), 79(30)
13	2220	(*Z*)-Oleic Acid, TMS derivative	0.87	0.36	117(100), 75(87), 73(83), 339(66), 129(66), 55(48), 145(35), 96(33), 43(27), 84(26)
14	2248	Stearic acid, TMS derivative	1.11	0.65	117(100), 73(71), 341(69), 132(55), 75(50), 129(35), 145(32), 43(22), 342(18), 55(18
15	2606	1-Monopalmitin, 2TMS derivative	0.27	n.d.	371(100), 372(29), 73(28), 147(21), 57(15), 239(15), 129(15), 43(13), 203(13), 71(10)

Table S2. Validation parameters for phenolic compounds.

	RT (min)	Wavelenght (nm)	Concentration range (mg/L)	Slope	Intercept	Coefficient of determination (R^2)	LOD (mg/L)	LOQ (mg/L)
**Gallic acid**	8.41	220	0.1–5	130811	−5036.1	0.9996	0.01	0.02
**Protocatechuic acid**	14.11	220	0.1–5	118500	−3875,6	0.9998	0.01	0.02
***p*-Hydroxybenzoic acid**	20.39	220	0.1–5	42638	−1313.8	0.9997	0.01	0.04
**Caffeic acid**	26.25	320	0.1–5	118614	−4165.4	0.9997	0,01	0.02
**Ferulic acid**	47.56	320	0.1–5	138722	−5417.1	0.9995	0.02	0.05
**Quercetin**	51.09	220	0.1–5	47496	−2330.5	0.998	0.02	0.06
**Rutin**	51.63	220	0.1–5	42271	−2781.8	0.994	0.01	0.02
**Myricetin**	56.24	220	0.1–5	70498	−5113.6	0.9995	0.01	0.03

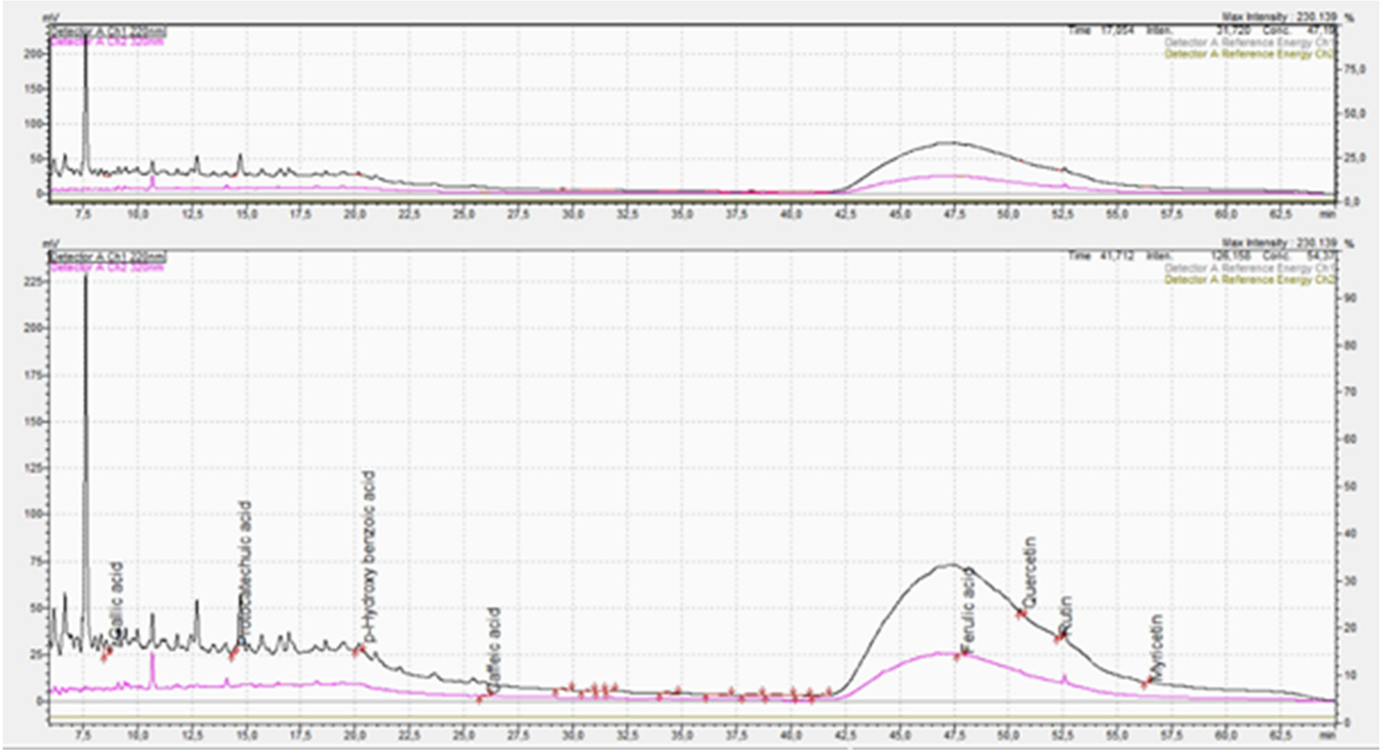

Figure S1. HPLC chromatograms of golden chanterelle aqueous extracts for extration at 70 °C, at 220 (black line) and 320 nm (pink line). Identified and quantified phenolic compounds in order of appearance with specified retention times: gallic acid (8.82 min), protocatechuic acid (14.27 min), *p*-hydroxybenzoic acid (20.58 min), caffeic acid (25.26 min), ferulic acid (47.32 min), quercetin (50.58 min), rutin (52.57 min) and myricetin (55.84 min).
